# Managing the Quality of Experience in the Multimedia Internet of Things: A Layered-Based Approach †

**DOI:** 10.3390/s16122057

**Published:** 2016-12-02

**Authors:** Alessandro Floris, Luigi Atzori

**Affiliations:** Department of Electrical and Electronic Engineering, University of Cagliari, 09123 Cagliari, Italy; l.atzori@ieee.org

**Keywords:** Internet of Things, Quality of Experience, multimedia

## Abstract

This paper addresses the issue of evaluating the Quality of Experience (QoE) for Internet of Things (IoT) applications, with particular attention to the case where multimedia content is involved. A layered IoT architecture is firstly analyzed to understand which QoE influence factors have to be considered in relevant application scenarios. We then introduce the concept of Multimedia IoT (MIoT) and define a layered QoE model aimed at evaluating and combining the contributions of each influence factor to estimate the overall QoE in MIoT applications. Finally, we present a use case related to the remote monitoring of vehicles during driving practices, which is used to validate the proposed layered model, and we discuss a second use case for smart surveillance, to emphasize the generality of the proposed framework. The effectiveness in evaluating classes of influence factors separately is demonstrated.

## 1. Introduction

The Internet of Things (IoT) is a world-wide network of interconnected objects, uniquely addressable, based on standard communication protocols [1]. Since its conception at the beginning of last decade, it has evolved by incorporating more and more technologies so that different types of devices are part of it: from RFID tags to sensors, from simple actuators to complex wireless sensors networks, from connected cars to wearables. With all of these kinds of objects, IoT covers many different domains of utilization, and several applications exist with heterogeneous requirements and purposes.

Briefly, IoT will be populated by an immense number of devices. Such devices will adopt heterogeneous technologies and standards and will have unequal capabilities in terms of processing, communication and energy availability. However, they will provide services integrated in critical applications involving the continuous monitoring and control of the physical environments in which humans live and operate.

Size, heterogeneity, the criticalness of the envisioned applications and the limitations of the resources of the components make the IoT a very complex environment, rich with opportunities and threats. It is unlikely that even the most advanced of the IoT devices will be able to survive and operate effectively in such a context, individually. IoT platforms have then the objective to combine and control the flows of traffic and signals received from different objects, processing in real time and offline the data and take actions, which will impact at the end (hopefully in a positive way) the quality of life of the humans.

In this scenario, the evaluation of the performance of IoT applications is becoming more and more important as relevant deployments are ubiquitously present in our everyday life activities. However, the plethora of IoT applications is quite vast, so that giving some guidelines on how to conduct this evaluation is very complex in a fast changing setting. Some studies related to this issue have been conducted with reference to the QoS (Quality of Service) evaluation models for IoT applications. However, there are no efforts in the direction of evaluating the QoE (Quality of Experience), which aims at assessing how end-users subjectively perceive the quality of an application or a service. Being user-centric, the QoE provides a more holistic understanding of the system’s influence factors with respect to the technology-centric measures of the QoS approach. Being closer to the user perspective, it better provides indications of to what extent the applications will be used by the users and what will be the real impact on the human quality of life.

Justified by these considerations, this paper focuses on the issue of evaluation and management of QoE in IoT multimedia applications. The following are the major contributions:

- We analyzed a reference IoT architecture to provide guidelines on which influence factors should be considered and measured for the evaluation and management of the QoE in different application scenarios.
- We introduced the concept of Multimedia IoT (MIoT) in order to focus on the applications in which people are involved as the end-users of the multimedia content provided by the MIoT applications.
- We defined a layered QoE management framework aimed at evaluating and controlling the contributions of each influence factor to estimate the overall QoE in MIoT applications.
- As a practical use case, we implemented a vehicular MIoT application that has been used to conduct subjective quality assessments to verify the applicability of the proposed approach.
- We presented and discussed a second use case for smart surveillance, to emphasize the generality of the proposed framework.

In Section 2, a background on IoT architectures, QoE models for IoT and layered QoE models is provided. In Section 3, we provide a classification of IoT applications and define the concept of Multimedia IoT. Section 4 presents the proposed layered QoE management framework for MIoT applications, whereas in Section 5, two use cases are discussed for emphasizing the generality of the proposed layered model. Finally, Section 6 concludes the article.

## 2. Background

The first subsection is devoted to the presentation of an IoT layered model, which is frequently adopted for the development of cloud-based platforms. The second subsection reviews the QoE definitions and provides an analysis of the past works that have touched the topic of QoE evaluation for IoT platforms, as well as those based on the layered approach for QoE modeling.

### 2.1. IoT Reference Model

The IoT refers to a network of interconnected objects that are able to acquire information from the physical world and to make this information available on the Internet. It is possible to basically identify three generations of IoT on the basis of the involved technologies [1]. The first one was developed around RFID tags, which were typically used for monitoring, logistics and tracking applications. The second generation was enhanced by sensors and actuators, which permitted acquiring various physical characteristics from the real world and brought to birth of Wireless Sensor Networks (WSN), which consist of a certain number of sensing and/or actuating nodes communicating in a wireless multi-hop fashion [2]. The third (and current) IoT generation is mostly developed around the association of a virtual object to each physical object. The virtual object has the role of virtualizing the functionalities of the physical object, which is then part of the IoT. Everyday physical objects become “smart” objects and are integrated within the global cyber-physical infrastructure [3]. Furthermore, the storage of the data and running of the application have been moved to the cloud.

From the second generation forward, multimedia devices have been playing a more and more important role in the IoT as the physical devices augmented with the virtual counterparts have become more powerful in the generation, processing and transmission of multimedia data.

**Figure 1:**
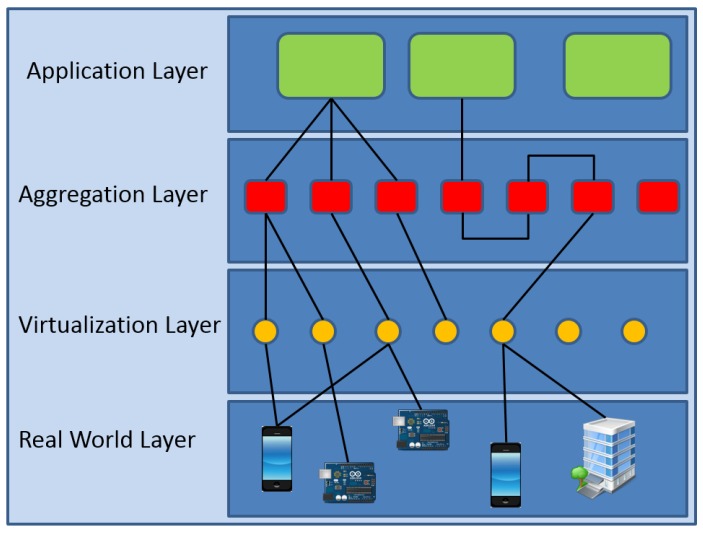
The considered four-layer model for IoT architectures.

Even if there is not a reference IoT architecture, we may say that a general four-layer model is generally followed as illustrated in Figure 1. A major feature of this model is the use of the virtual object, which is a digital representation of the physical object. One specific implementation that follows this architecture is the iCore solution [4], which exploits and leverages the adaptation capabilities offered by the platform in terms of virtualization, aggregation and abstraction properties.

The following are the functionalities of the different layers:

- Real-world layer: refers to the Real-World Objects (RWOs), i.e., the physical sensing devices that acquire the information that will be used by the IoT application;
- Virtualization layer: creates the Virtual Objects (VOs), which virtualize the functionalities of the associated RWOs;
- Aggregation layer: different VOs can be combined in order to create Composite Virtual Objects (CVOs) capable of providing a determined service that a single VO cannot accomplish;
- Application layer: plans and understands what requested services are needed by the IoT application. As such, it determines the strictly needed Service Level Agreement (SLA) that the platform is to execute by means of its CVO (and VO); this layer also exports all of the system’s functionalities to the final user.

A specific implementation of an IoT solution that follows this model is [5]. With regard to quality assurance, the iCore architecture considers an SLA assurance function at the CVO level. Based on available policies, this function may observe if the matching CVO for a certain service execution request is deployable and executable based on the current and predicted availability of resources for SLA fulfillment. However, a module for evaluating the final quality provided to the end-user of the IoT application is not considered.

It is worth mentioning that since 2008, the MPEG group has been working on a standard called MPEG-V (ISO/IEC (International Organization for Standardization/International Electrotechnical Commission) 23005) [6,7,8], which aims to define a standard way of representing the data from sensors and actuators in the real world, as well as a common interface to virtual worlds. As a result, each proprietary virtual world will not be isolated from the other virtual worlds. In particular, MPEG-V defines the XML schema for the information about sensor/actuator capabilities, user preferences, sensed information and data types used by actuator commands. These data type elements correspond to descriptions of devices and messages for “talking to” and “adapting to” either devices or services in the IoT. In addition, the Part16 of MPEG Exploration (called Internet of Media Things and Wearables (IoMT & W)) is trying to understand IoT requirements that are not yet satisfied by MPEG-V [9]. Therefore, in this draft, the media-centric Internet of Things is defined, as “the collection of interfaces, protocols and associated media-related information representations that enable advanced services and applications based on human to device and device to device interaction in physical and virtual environments”. Furthermore, the definition of media thing is introduced, as “a Thing with at least one of audio/visual sensing and actuating capabilities”. The scope of IoMT & W is to standardize a set of interfaces, protocols and associated media-related information representations. However, to date, only media thing requirements and some use cases have been defined.

Although the architecture proposed by MPEG-V is of interest, especially with the integration of the media things, in this work, we mainly refer to the four-layer model in Figure 1, since it is a general architecture that can be used as a reference for any type of IoT application. Furthermore, MPEG-V does not consider any module for the evaluation of the quality provided by its proposed framework. However, the concepts defined by the MPEG-V standard will also be considered for the definition of the proposed QoE management framework.

### 2.2. QoE Evaluation and Management

In the Qualinet White Paper on Definitions of Quality of Experience [10], the QoE is defined as “the degree of delight or annoyance of the user of an application or service. It results from the fulfillment of his or her expectations with respect to the utility and/or enjoyment of the application or service in the light of the user’s personality and current state”. Such a definition of QoE is valid for general multimedia applications and services; therefore, it can also be extended to IoT applications. However, currently, there is no reference model for evaluating the QoE of IoT applications, probably due to the lack of a reference IoT architecture and to the different requirements of IoT applications belonging to different domains of utilization. Indeed, there are only a few works that have started addressing this issue.

In [11], the authors focused on the perceived quality in actuators connected to the IoT. They developed a test bed that consisted of an electro-mechanical arm controlled over a packet switched unreliable link. The experiment required users to direct a fixed laser attached to the fixed arm’s grabber towards a set of targets. The experimental factors were the average one-way delay, the packet loss and the number of degrees of freedom of the arm. From the subjective quality results, in terms of the Mean Opinion Score (MOS), the authors defined a parametric QoE model that estimates the QoE as a function of the considered experimental parameters. While [11] focused on a specific IoT application, other studies proposed a QoE model for general IoT systems. Due to the lack of a reference IoT architecture, each work proposes its own vision of the IoT and of the consequent quality evaluation model. In [12], the authors defined the Cognitive Internet of Things (CIoT), a new network paradigm where physical and virtual things or objects are interconnected and behave as agents, with minimum human intervention. The CIoT framework measures three different qualities: Quality of Data (QoD), i.e., the quality of sensed data; Quality of Information (QoI), i.e., the information that meets a specific user’s need for a specific time, place and social setting; QoE, focused on factors belonging to four levels: access, communication, computation and application. While [12] considered both QoS and QoE parameters, [13,14] only focused on QoS evaluation. In fact, the authors defined an IoT architecture composed of three layers (i.e., sensing, networking and application), and for each of these, a monitoring module manages the resource allocation as a function of the measured QoS metrics: information accuracy, sensing precision and energy consumption at the first layer; bandwidth, delay, throughput and coverage at the network layer; service performance cost, performance time, load and reliability at the application layer. Although QoS parameters are important for the performance evaluation of an IoT platform, they should be considered as a function of the quality perceived by the end-user and not only as a fulfillment of SLAs.

However, it can be noticed that these works follow a layered approach to consider different influence factors within each layer. In fact, many different QoE influence factors exist and are defined as “any characteristic of a user, system, service, application, or context whose actual state or setting may have influence on the QoE for the user” [10]. In the literature, several works grouped “related” QoE influence factors into well-defined domains, spaces or layers. For example, the ARCU (Application Resource Context User) proposal models QoE influence factors as falling into one of four multi-dimensional spaces: application, resource, context and user [15]. Points from the ARCU space are then mapped (with objective models or subjective evaluations) to points in a QoE space, which is composed of dimensions representing different quantitative and qualitative quality metrics, which can be perceived by an end-user (e.g., MOS, ease-of-use, efficiency, comfort). The theoretical approach defined by the ARCU model was used in [16] to define an operational approach: the QoE layered model. The six layers defined by this model basically respect the spaces defined by the ARCU model. However, two spaces were further modeled: the context space is represented by the interface and the context layers, while the user space is represented by the human and the user layers. In [17], the hourglass model is proposed, which is composed of four layers: QoS; Quality of Delivery (QoD), i.e., the quality of data delivered by both the application and the network; quality of presentation; QoE. As is common for layered approaches, each quality estimation is in turn implemented utilizing the quality estimation provided by the previous bottom layers. Therefore, starting from the QoS, the authors defined the functions brought to the estimation of the QoE. We also investigated in a past study the utilization of a layered approach for modeling the QoE in multimedia services [18]. The objective was to integrate the results of different modeling activities in order to solve un-interoperability issues between different models.

The layered approach was also used in [19] to model their proposed QoE management framework into the data acquisition, monitoring and control levels. Besides providing a thorough analysis of the most relevant QoE management works available in the literature, the authors proposed a generic QoS/QoE framework for enabling quality control in packet-switched networks. They mainly focused on the management component of the framework to show how data collected from a network can be refined into knowledge of quality perceived by users and how to take corrective measures when necessary. Although QoE management frameworks have been also proposed for cloud applications [20] and future Internet architectures [21], to the best of the authors’ knowledge, no studies have focused on QoE management for IoT applications, which is the objective of this paper. We aim at defining a specific framework for the evaluation and management of the QoE provided by IoT applications and specifically of multimedia IoT applications; on the basis of a layered IoT architecture, we consider at each layer the most relevant QoE influence factors and combine them at the higher layers to provide a measure of the overall QoE provided by the IoT application to the end-users.

## 3. Preliminary Analysis

In this section, we first analyze the variegate scenarios of IoT applications where multimedia data have a major role and the user is the final consumer of the offered services. Then, we define the multimedia IoT scenario that is used in the following for our QoE management framework.

### 3.1. Multimedia IoT Applications and QoE

A huge number of IoT applications has been developed for different objectives and services. Generally, as in [1,2,3,22], IoT applications are classified on the basis of the domain of utilization.

The main domains are:

- Healthcare: remote patient health real-time monitoring, patient-flow monitoring, identification and authentication of patients and staff and automatic medical inventory management are examples of healthcare applications.
- Personal and social: the user is enabled to interact with other people to maintain and build social relationships. Applications concern the automatic update of information about social activities in social networks and search engines for things with RFID to prevent losses and thefts.
- Smart home/smart building: instrumenting buildings with advanced IoT technologies brings to adaptable rooms heating and lighting, monitoring and alarm systems, smart household appliances and the optimization of power consumption costs (together with the smart grid).
- Smart city: IoT applications concern the optimization of physical city infrastructure and quality of life for citizens, smart parking and pollution monitoring.
- Smart grid and smart metering: the energy consumption can be efficiently monitored in a smart home or in a small office or building.
- Smart environment: monitoring and transmission of critical parameters of the environment, access to critical areas dangerous for human operators (volcanoes, etc.) and natural disaster monitoring and detection.
- Transportation: smart vehicles equipped with sensors and actuators, together with WSN produce information for assisted driving and traffic monitoring applications; identification and monitoring of objects, people and animals in motion with tracking applications.
- Smart business and logistics: RFID technology used for inventory management and monitoring throughout supply/delivery chain, monitoring of real-time product availability and retrieval of products’ data after purchase.
- Security and surveillance: smart video cameras with automatic behavior analysis and event detection, ambient sensors, alarms and RFID for personal identification.

Not for all of these applications is the QoE management of interest, as the user is not always the recipient of the services. Indeed, if the application output is received by another system, then the performance of the application can be evaluated through a classical QoS-based approach. In fact, the objective of the QoE management activity is to have the whole control of the delivered service as perceived by the end-user to optimize the quality while controlling the utilized resources of each system component (in the IoT platform for the interest of this work). Additionally, in this work, we are interested to the evaluation of the QoE for applications where the multimedia content has a major role.

**Figure 2:**
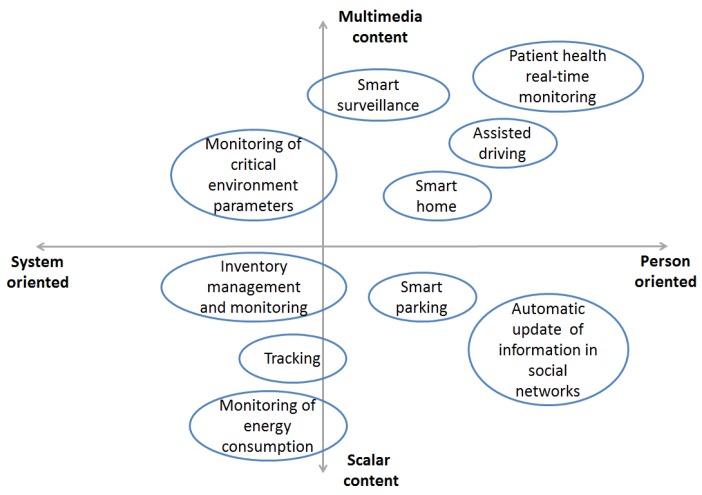
Classification of IoT applications on the basis of the type of information they manage (multimedia or scalar content) and of the type of recipient (person or system oriented).

QoE requirements can be very different with respect to the considered IoT application domain; furthermore, QoE requirements can also be different among IoT applications belonging to the same IoT domain. This is also shown in Figure 2, where we present the distribution of IoT applications that are representative for each IoT domain. These are placed on the picture on the basis of the type of information they manage (multimedia or scalar content) and the type of end-user orientation (person or system oriented). A person-oriented IoT application means that people have a fundamental role in the evaluation of the IoT application, since they are the end-users who benefit from the information and content provided by the IoT application. On the other hand, a system-oriented IoT application refers to those applications that automatically acquire, control and manage data in order to do specific tasks and make specific decisions. In these applications, human intervention and evaluation are not essential. Nonetheless, human participation must always be considered also for system-oriented applications, for which humans have the task of “smart” controllers and users.

### 3.2. The Multimedia Internet of Things

The emerging categories of IoT objects tend to be mobile, multi-sensorial and smart, such as wearable sensors, smartphones and smart vehicles, which more and more introduce multimedia content into the service chain. As already mentioned in Section 2, the IoT is often defined as a network of interconnected objects able to acquire information from the physical world and make this information available on the Internet. By adding the multimedia content, the Multimedia IoT (MIoT) can be defined as a “network of interconnected objects capable to acquire multimedia contents from the real world and/or present information in a multimedia way”. In the same way, multimedia objects can be defined as “objects capable to acquire multimedia contents from the physical world, being equipped with multimedia devices such as cameras and microphones”. This definition includes the IoMT & W concept previously mentioned, as the latter focuses on the devices that are capable of performing at least one audio/visual sensing or actuating action. However, the multimedia IoT refers to all of the layers in the IoT, where the multimedia flows are processed, analyzed, merged and stored in each layer for their respective purposes. It also includes the presentation layer where multimedia content can be used to present the relevant application results and control functions.

In the MIoT, we can distinguish three scenarios on the basis of the use of the multimedia content:

1. Multimedia as IoT input and output: the multimedia content is acquired by multimedia objects, and it is presented in a multimedia way by an IoT application;
2. Multimedia as IoT input: the multimedia content is acquired by multimedia objects, and it is used by an IoT application to provide a determined service;
3. Multimedia as IoT output: IoT objects acquire signals, data and information (scalar content) that are presented in a multimedia way (e.g., by using graphs, animations, etc.) by an IoT application.

**Figure 3:**
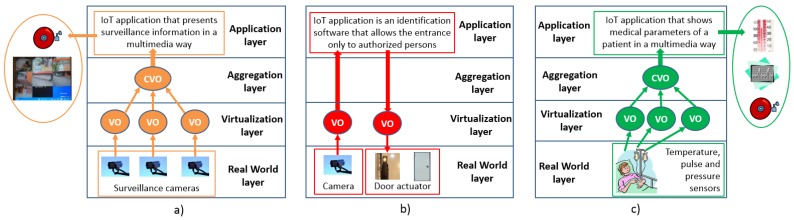
Multimedia IoT (MIoT) scenarios: (**a**) multimedia as IoT input and output: IoT application that presents in a multimedia way information acquired by multimedia objects; (**b**) multimedia as IoT input: IoT application that uses information acquired by multimedia objects; (**c**) multimedia as IoT output: IoT application that presents in a multimedia way information acquired by IoT objects (scalar content).

The first scenario is represented by the use case depicted in Figure 3a, where some surveillance cameras record images and audio of a place. This multimedia information is collected by an IoT application and presented in a multimedia way (images, videos, audios) in order to provide a remote security control service. Figure 3b illustrates the second scenario. A camera records some images of people who want to enter in a place where the entrance is allowed only to authorized persons. The images recorded by the camera are then sent to an IoT platform where an application is deployed that consists of an identification software that has to determine if the person recorded by the camera is authorized to enter to that place. Once the software identifies the person, a command is sent to the door actuator to open the door. Otherwise, the door stays closed, and access is denied. Finally, Figure 3c shows the last case. Various IoT objects measure some medical parameters of a patient (e.g., temperature, pulse, pressure), which are then collected by the IoT platform. This application has the task to present the status of the patient in a multimedia way, for example by using graphs, animation, alarms, etc.

## 4. Layered QoE Management Framework for MIoT Applications

As already analyzed in Section 2, there is not a reference model for evaluating the QoE of IoT (and then MIoT) applications, probably due to the lack of a reference IoT architecture and to the different requirements of IoT applications belonging to different domains of utilization. Our contribution in this direction is to leverage a layered-based approach for evaluating and controlling the QoE of MIoT applications, in which people are involved as the end-users of the multimedia content. We chose to follow a layered approach since the objective is to evaluate different categories of QoE influence factors in different layers and then to combine these measures in order to maximize the final QoE perceived by the user using the IoT application.

Layered approaches are widely used in networking for their functionality, such as the TCP/IP and OSI (Open Systems Interconnection) networking models. Within each layer, a well-defined set of network services is provided, which is in turn implemented utilizing the services provided by the previous layers. At the lower layers, services tend to be simple and efficient. As successively higher layers are considered, functions become gradually more complex. Following this layered approach, each layer must be defined together with its area of competence. In our case, each layer must model the quality provided by a certain IoT layer and must be able to be combined with its upper and lower layers; this feature is fundamental in order to build a model in which the outcome of a layer can be interpreted and then gradually enhanced by higher layers. For such a reason, interfaces between layers should be accurately defined.

**Figure 4:**
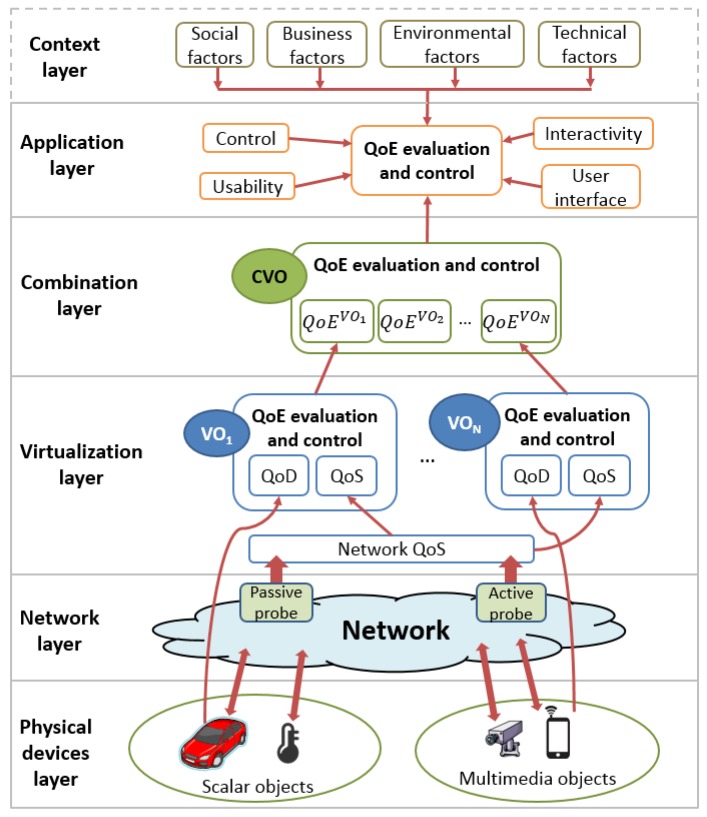
The proposed layered Quality of Experience (QoE) management framework.

The layered model allows for defining each layer singularly and independently from the others. Each layer focuses on a specific QoE domain (set of factors), so that the overall quality can be computed as a combination of all domains. The proposed model is meant to be as general as possible, so to be valid for, or at least adaptable to, any scenario for MIoT. On the basis of the four-layer IoT architecture presented in Section 2, we propose our layered QoE management framework as a set of modules that can be integrated in the IoT architecture and are capable of evaluating and controlling the QoE of the multimedia IoT applications. The proposed layered QoE management framework is shown in Figure 4 and is composed of five layers: the physical device layer, the network layer, the virtualization layer, the combination layer and the application layer. We consider the influence of the context layer on the QoE within the application layer.

### 4.1. Physical Device Layer

In this layer, the physical devices are considered, i.e., the RWOs. We distinguish between multimedia objects and scalar objects: the former are the objects capable of acquiring multimedia content, whereas the latter are the objects capable of acquiring scalar content. The information acquired by both of these two types of objects is sent to their associated VOs through the network on the basis of the network interface of the RWO. This layer focuses on the quality of the data (QoD) acquired by the physical devices and taken into account at the virtualization layer. For example, the performance of a GPS receiver can be determined by measuring the accuracy and precision of the acquired position, which is the QoD. Furthermore, the QoD of a video sequence recorded by a camera accounts for the image resolution, the frames per second and the video coding distortion. Indeed, as the VO is the digital counterpart of the physical device, in our view, it also takes care of managing the QoD according to the registered physical device characteristics.

### 4.2. Network Layer

The network layer accounts for the network infrastructure on which the IoT platform is based. In this layer, typical QoS parameters capable of measuring the performance of data transmission are considered, such as delay, bandwidth, packet loss, jitter, etc. The network’s performance can be measured using passive or active probes. Passive probes are non-intrusive network monitoring agents that collect data without causing additional traffic overhead on the network, whereas active probes send dedicated measurement packets to benchmark the network performance. The measurements collected by the probes are sent to the network QoS module of the Virtualization layer, whose role is the execution of statistical analysis on the data collected for evaluating the quality of the network.

### 4.3. Virtualization Layer

In the virtualization layer, the VOs are created, which are processes typically instantiated in the cloud. Each VO is provided with a VO template that can be associated with one or more physical devices of which it virtualizes the functionalities. In the IoT cloud platform, the users have to choose a VO template to be associated with their physical devices, if existing, or they can create a proper VO template. In fact, each VO template should be defined by the brand of the physical device and is basically composed of a description of the device and a list of the device’s parameters corresponding to the scalar and/or multimedia contents that can be acquired by the physical device. Otherwise, the owner of the object can define a proper VO template for his/her device. Furthermore, if the object is an analog transducer, the IEEE 1451.4 Transducer Electronic Data Sheets (TEDS) [23] can be used as the VO. In fact, this IEEE standard contains the critical information needed by an instrument or measurement system to identify, characterize, interface and properly use the signal from an analog sensor. The TEDS can reside in embedded memory within the analog transducer or a virtual TEDS can exist as a separate file, downloadable from the Internet. This concept of virtual TEDS is the equivalent of the VO for the IoT platform.

The network QoS module has the task to receive the measures collected by the network probes and to create statistical analysis about the network traffic for evaluating the QoS provided by the network. Each VO can use this network information by considering the network parameters of interest for estimating the QoS of the network. As a consequence of network impairments, the data collected by the physical device may be impaired. For example, network delay may create latency problems for real-time applications, whereas packet losses may cause gaps in the data. Therefore, we consider QoS as an influence factor for the QoD acquired by a physical device (PD):

(1) $$QoD^{PD}=f_{1}\left(QoS\right).$$

As shown in Figure 4, each VO is provided with a QoE evaluation and control module, which has to evaluate and control the overall QoE provided by the VO. The QoE is evaluated by considering the influence of both QoD and QoS parameters on the QoE perceived by the users, so that:

(2) $$QoE^{VO}=f_{2}\left(QoD^{PD}\right)=f_{2}\left(f_{1}\left(QoS\right)\right).$$

This can be done by considering quality models able to map objective measures (QoS and QoD) with the subjective perceptions of the users in terms of the MOS. If specific models do not exist, objective metrics can be used or subjective assessments should be conducted to define the needed QoE mapping models. QoS parameters are estimated on the basis of the information provided by the network QoS module by considering only the network parameters of interest for the VO. On the other hand, the QoD refers to the quality of the content acquired by the physical device. For example, the QoE of a video acquired by a camera could be a function of the video bitrate, resolution, codec and quantization parameters used for acquiring and encoding the video; the QoE of a temperature sensor could be a function of the precision and accuracy of the temperature sensor. The device parameters to be considered for evaluating the QoE of each object are listed on its VO template.

The QoE evaluation and control module is also capable of controlling the QoE of the VO, which means that it can order the physical device associated with the VO to modify the value of its parameters if possible; for example, for the case of a camera, it can order to reduce or increase the resolution or bitrate used for acquiring the video. The modification of these parameters brings a modification of the QoE perceived by the users.

### 4.4. Combination Layer

In the combination layer, the CVOs are created. As said before, the CVO is composed of various VOs with the aim of combining their functionalities to provide a determined service, which a single VO cannot accomplish. Furthermore, the CVO has to evaluate the overall QoE provided by the VOs, which are composing it. Therefore, the CVO is provided with a QoE evaluation and control module that has to properly combine the influence of each VO on the QoE perceived by the users:

(3) $$QoE^{CVO}=f_{3}\left(QoE^{VO_{1}},QoE^{VO_{2}},\ldots ,QoE^{VO_{N}}\right)=f_{3}\left(f_{2}\left(QoD^{PD_{1}}\right),f_{2}\left(QoD^{PD_{2}}\right),\ldots ,f_{2}\left(QoD^{PD_{3}}\right)\right),$$

where for each physical device, the QoD is a function of the QoS, as in Equation (1).

For example, the QoE can be estimated with a mathematical model that combines and weighs each single quality contribution provided by the VOs in order to achieve the maximum quality for the requested service. The QoE evaluation and control module is also capable of controlling the QoE of the CVO, which means that, on the basis of the single contribute provided by each VO, it can order the VOs to modify the value of their parameters, if possible, to maximize the QoE. This order is then propagated from the VOs to their physical devices.

### 4.5. Application Layer

The application layer considers the QoE provided by MIoT applications in terms of control, interactivity, presentation and usability. In fact, independently from the quality of the multimedia content provided by the application, the quality perceived by the end-users is also influenced by the presentation of the contents to the user, the application interface, the degree of interactivity and the controllability and usability of the application. The QoE evaluation and control module evaluates the influence of these parameters on the QoE. Furthermore, it also considers the influence of the context factors on the application. The context of use concerns many different factors, such as: the type of device on which the application is used (technical factors); the people with which the end-user consumes the application (social factors); the application costs (business factors); and the place where the application is used (environmental factors). Context parameters can be determined by the application through information provided by the users by means of their user profiles, surveys and/or hardware/software able to automatically determine information related to the device in which the application is running, the place where the user is located (for example with a GPS sensor) and other factors. Therefore, the final QoE perceived by the users can be expressed as:

(4) $$QoE^{App}=f_{4}\left(QoE^{CVO},AppFactors,ContextFactors\right).$$

where *AppFactors* and *ContextFactors* are the influence factors concerning the application and the context of use, respectively. To evaluate how these influence factors impact the QoE of the users, subjective assessments have to be conducted.

## 5. Analysis of Use Cases

In this section, we provide two examples of the application of the proposed QoE management framework and its layered approach on real MIoT applications. The objective is to show how quality models can be defined for the VOs as a function of QoS and QoD parameters and how these QoE models can be combined by the CVO for evaluating the overall QoE of an MIoT application. For one of the investigated applications, the MIoT vehicle application, we conducted a subjective quality assessment for validating the resulting QoE models. The same process can be followed for the second considered use case, i.e., the smart surveillance application.

### 5.1. MIoT Vehicle Application

We have developed an IoT multimedia system aimed at remote monitoring and tutoring practitioners when driving a car. The objective of this application is to show in a multimedia way and in real time the state and the position of the vehicles during driving lessons together with a video showing a view of the roads traveled by these vehicles. In this way, the instructors can remotely monitor and evaluate the results achieved by the practitioners.

**Figure 5:**
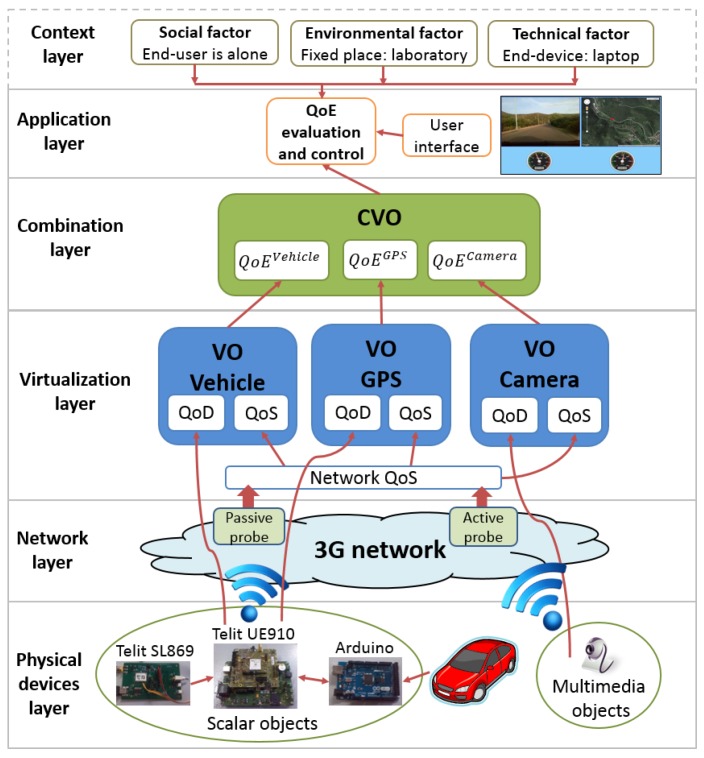
Framework of the MIoT vehicle application for remote tutoring for driving school lessons.

Figure 5 shows the proposed QoE framework applied to this specific vehicle application. The physical device layer accounts for the physical devices acquiring the information needed by the application; these are an Arduino Mega 2560 [24], the Telit UE910-EUR and SL869 modules [25], and a camera. The Arduino Mega 2560 is connected to the On-Board Diagnostic interface (OBD-II) of the vehicle and is able to acquire vehicle parameters. For this use case, we acquired the speed and revolutions per minute (rpm) parameters. The Telit SL869 module is provided with a Global Positioning System (GPS), which acquires the position coordinates of the vehicle with the current date and time. The vehicle data collected by the Arduino and the SL869 are sent to the IoT platform (which is in the cloud) by the UE910-EUR, which is provided with 3G connection and acts as a network interface between the sensors and the IoT platform. Simultaneously, the camera installed in the vehicle cabin records and sends to the IoT platform the video sequences of the road traveled by the vehicle through the 3G network.

In the network layer, the performance of the 3G network used by the physical devices for communicating with the VOs is taken into account. In the virtualization layer, three VOs are created: the VO of the vehicle receives the vehicle speed and rpm data; the VO of the GPS receives the coordinates of the vehicle position; the VO of the camera receives the videos recorded by the camera.

**Figure 6:**
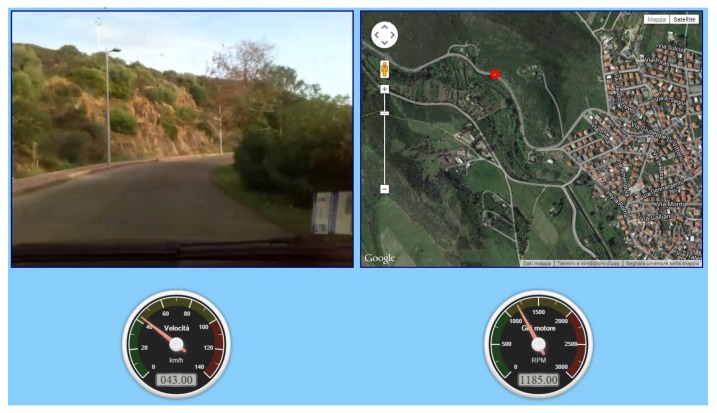
Screenshot of the web MIoT application with analog speed and rpm indicators.

**Figure 7:**
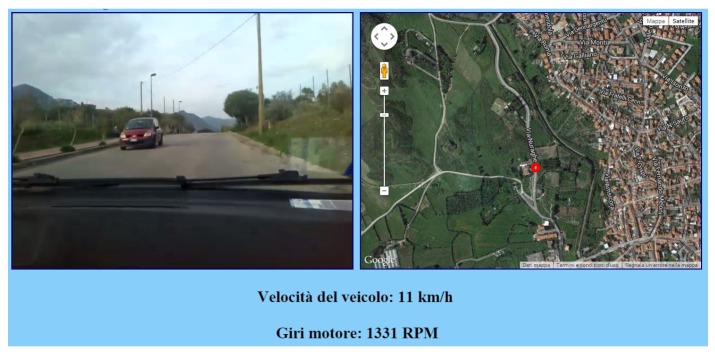
Screenshot of the web MIoT application with digital speed and rpm indicators.

In the combination layer, a CVO is created that receives the data acquired by the three VOs; herein, the vehicle parameters, the GPS coordinates and the video sequences (the images recorded by the camera are tagged with the date and time) are analyzed and synchronized. Finally, in the application layer, the combined information provided by the CVO is shown in a multimedia way to the users on a web application. Specifically, the web MIoT application shows in a web page a video of the road traveled by the vehicle, a map in which is shown the vehicle position (highlighted with a moving red circle) and the speed and rpm of the vehicle. Figure 6 and Figure 7 show a screenshot of the web MIoT application where speed and rpm parameters are shown to the users with analog and digital indicators, respectively.

#### 5.1.1. Test Conditions

We defined some Test Conditions (TCs), summarized in Table 1, for conducting a subjective quality assessment and determining the QoE of the vehicle MIoT application.

The parameters chosen to create the TCs are:

- Video bitrate: it is the QoD parameter for the video content recorded by the camera. Six different videos were recorded and encoded at two different bitrates: 700 kbps for low video quality and 1.5 Mbps for high video quality. The videos were encoded with the MPEG-4 codec at a resolution of 640 × 480 pixels and a frame rate of 24 fps.
- Accuracy of vehicle data: it is the amount of data correctly acquired by the physical devices. In this case, it is the QoD parameter for the Arduino and GPS devices. We artificially introduced errors in the data acquisition by modifying the value for 25% of the data acquired by these two devices. With regard to speed and rpm, modified values were replaced with random values in the range ±100% with respect to the acquired value, whereas for the vehicle position, we modified the acquired coordinates to shift the vehicle position for a maximum of 10 m with respect to the acquired position (again, the position was generated randomly in the distance range of 0–10 m). Modified parameters were shown randomly to the user (on average, one out of four values were modified) and could also happen sequentially.
- Network delay: it is the QoS parameter of the 3G network used by the UE910 module (we do not consider network delay for the network used by the camera in order to obtain a desynchronization between the video sequences and the vehicle data). In some TCs, a network delay of 3 s involves the web application showing delayed values of GPS, speed and rpm data with respect to the video sequences. Network delays are introduced in a way that the user can notice them, for instance when the vehicle is starting or stopping.
- Data presentation: it is an application influence factor. Vehicle data (speed and rpm) are presented to the users with both analog and digital indicators, as in Figure 6 and Figure 7.

Furthermore, with regard to context influence factors, the web application is consumed by the user on a laptop (technical factor), alone (social factor) and in a laboratory for a test session (environmental factor).

#### 5.1.2. QoE Evaluation

In the conducted subjective assessment, 24 people, 20 males and 4 females, were asked to rate the 12 TCs summarized in Table 1. The assessment started with a pre-test phase, during which the user was provided with short written instructions and with a brief detailed oral explanation. Furthermore, some TCs were shown in order to give each user an overview of the video qualities and data corruptions involved in the experiment. The parameters that the user had to consider in the subjective evaluations were: the video quality; the synchronization and accuracy of the vehicle data with respect to the video; the overall presentation of the vehicle data.

For subjective tests, the single-stimulus Absolute Category Rating (ACR) method has been adopted [26], which consists of presenting the TCs one at a time and allowing for their independent evaluation on a category scale. The following five-level ITU scale for rating overall quality has been used: 5 stars (excellent), 4 stars (good), 3 stars (fair), 2 stars (poor), 1 star (bad). The device employed for the subjective quality assessment was a laptop with an LCD display with a resolution of 1440 × 900 pixels and an aspect ratio of 16:9. The web MIoT application was displayed on the Google Chrome browser in full-screen mode while each subject was wearing earphones. Each TC lasted for 40 s, and the assessment has been carried out in a 15 min-long session. A rating page was automatically displayed after the end of each TC, where each subject had to select a discrete value between one and five indicating the overall quality of the TC just shown. The voting phase was not time limited, and after each vote, the subjects were asked to confirm their choice. Upon confirmation, a 10-s pause was imposed, then the next TC would be automatically activated. Such an automatic procedure was iterated for all 12 TCs. The presentation order of the TCs has been randomized for each subject.

**Figure 8:**
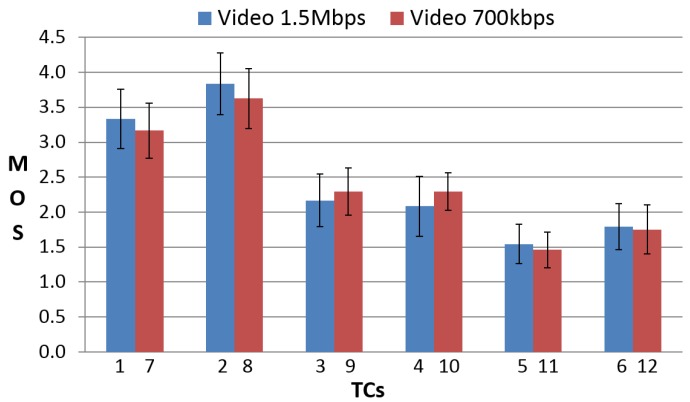
Mean Opinion Score (MOS) results with the 95% CI comparing low and high video qualities being equal in the other conditions.

**Figure 9:**
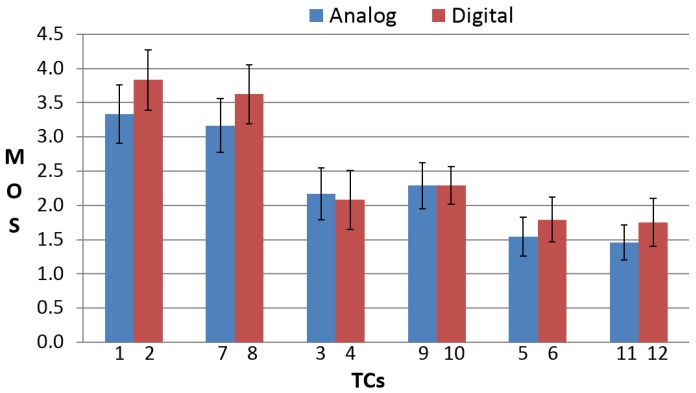
MOS results with 95% CI comparing analog and digital presentation of vehicle parameters being equal in the other conditions.

The MOS was computed from subjective results for each TC, together with the 95% Confidence Interval (CI). Figure 8 shows MOS results comparing low and high video qualities being equal in the other conditions, whereas Figure 9 shows MOS results comparing the analog and digital presentation of vehicle parameters being equal in the other conditions.

From Figure 8, MOS results show that video quality does not influence very much the QoE of the vehicle MIoT application. In fact, being equal in the other conditions, the MOS for low and high video quality have more or less the same value. This means that, in this specific application, the end-users are more interested in the accuracy and synchronization of the vehicle data than in the quality of the video sequences. With regard to the presentation of the vehicle data, MOS for digital data presentation have, in the majority of cases, slightly higher values than MOS for analog data presentation, as shown in Figure 9. In fact, except for TCs concerning delayed data (TC3, TC4, TC9, TC10), the users preferred digital data visualization. This could be due to the fact that digital indicators are more precise than analog indicators since the user can know the exact values of the vehicle speed and rpm. Furthermore, MOS results show that the most annoying TCs are those where wrong vehicle data are displayed (TC5, TC6, TC11, TC12). Then, the accuracy of the data is the most relevant parameter considered by the end-users. TCs with delayed data also annoy the end-users, but to a lesser extent (TC3, TC4, TC9, TC10). The evident result is that from this vehicle MIoT application, the end-users expect the displaying of accurate and synchronized vehicle data; otherwise, they were annoyed. On the other hand, video quality and data presentation do not have a great influence on the QoE.

#### 5.1.3. QoE Modeling

As discussed in Section 4, each VO is provided with a QoE evaluation module that has the role of evaluating the QoE of the data received by its associated physical device as a function of the QoD and QoS parameters. Specifically, in this case, the VO of the camera evaluates the QoE as a function of the video bitrate (Rate), whereas the VOs of the vehicle and the GPS evaluate the QoE in function of the accuracy (Acc) of vehicle data and the delay (Delay) of the 3G network.

The subjective ratings provided by the users have been divided into two datasets: a training dataset composed of the ratings provided by the first 12 subjects and a validation dataset composed of the ratings provided by the last 12 subjects. From the training dataset, we computed the $MOS_{T}$ values, while from the validation dataset, we computed the $MOS_{V}$ values. For modeling and mapping the QoE as a function of the QoD and QoS parameters, we computed a linear (l) and a logarithmic (nonlinear (nl)) regression between inputs (QoS and QoD) and output data (QoE, i.e., $MOS_{T}$). We used the linear approach because it is a simple approach that may provide good results with a small dataset like ours and the logarithmic/exponential approach because it is usually used for modeling the quality perception with regard to QoS [27,28].

(5) $$QoE_{l}^{Camera}=a_{1l}\times Rate+K_{1l}$$

(6) $$QoE_{l}^{Vehicle}=a_{2l}\times Acc+K_{2l}+a_{3l}\times Delay+K_{3l}$$

(7) $$QoE_{l}^{GPS}=a_{2l}\times Acc+K_{2l}+a_{3l}\times Delay+K_{3l}$$

(8) $$QoE_{nl}^{Camera}=K_{1nl}\times {\left(a_{1nl}\right)}^{Rate}$$

(9) $$QoE_{nl}^{Vehicle}=K_{2nl}\times {\left(a_{2nl}\right)}^{Acc}\times K_{3nl}\times {\left(a_{3nl}\right)}^{Delay}$$

(10) $$QoE_{nl}^{GPS}=K_{2nl}\times {\left(a_{2nl}\right)}^{Acc}\times K_{3nl}\times {\left(a_{3nl}\right)}^{Delay}$$

The equations in (5)–(10) are the models that describe the relation between the QoS and QoD parameters and the QoE of the VOs.

Two regressions have been computed: one for the TCs considering digital data presentation and one for the TCs considering analog data presentation. The coefficients obtained by computing these regressions are summarized respectively in Table 2 and Table 3 and are valid for the equations in (5)–(10).

In the previous section, from the analysis of MOS results, we concluded that video quality does not influence the QoE, and the data regressions confirm this result; in fact, the linear and nonlinear coefficient values ($a_{1l}$ and $a_{1nl}$) for the rate parameter in Equations (5) and (8) are zero and one, respectively, i.e., the identity elements of the addition and multiplication operations. Therefore, the value of the Rate does not influence the QoE. However, this could also be due to the limited and closer values of bitrates chosen for coding the video.

In the combination layer, the QoE model of the CVO combines the QoE provided by each VO in order to evaluate the overall QoE. Therefore, the composite linear and nonlinear QoE models of the CVO are as follows:

(11) $$\begin{array}{cc} QoE_{l}^{CVO} & =a_{1l}\times Rate+K_{1l}+a_{2l}\times Acc+K_{2l}+a_{3l}\times Delay+K_{3l} \end{array}$$

(12) $$\begin{array}{cc} QoE_{nl}^{CVO} & =K_{1nl}\times {\left(a_{1nl}\right)}^{Rate}\times K_{2nl}\times {\left(a_{2nl}\right)}^{Acc}\times K_{3nl}\times {\left(a_{3nl}\right)}^{Delay} \end{array}$$

Since we concluded that the Rate parameter does not influence the QoE, these equations become:

(13) $$QoE_{l}^{CVO}=a_{2l}\times Acc+K_{2l}+a_{3l}\times Delay+K_{3l}$$

(14) $$QoE_{nl}^{CVO}=K_{2nl}\times {\left(a_{2nl}\right)}^{Acc}\times K_{3nl}\times {\left(a_{3nl}\right)}^{Delay}$$

Finally, at the application layer, the QoE evaluation and control module must decide which coefficients to use from Table 2 and Table 3 for Equations (11)–(14). This decision must be made according to the user interface of the application that can present vehicle data with digital or analog indicators. Table 4 and Table 5 summarize the Pearson correlation values computed between the $MOS_{V}$ values and the quality models defined in Equations (11)–(14) respectively with regard to the digital and analog data presentation. The Pearson correlation results are very high, especially for the linear model, which means that the proposed linear model is highly correlated with subjective evaluation and can be used to estimate and control the QoE of the vehicle MIoT applications. Furthermore, these results confirm the validity of the proposed layered QoE model, which allows combining the single QoE contributions in order to evaluate the overall QoE perceived by the users. We computed the Pearson correlation values for both the equations considering/not considering the Rate parameter for further demonstrating that the inclusion of this parameter in the model does not bring a better precision in quality prediction.

In a future implementation, the QoE evaluation and control module of the combination layer should automatically notice that the Rate parameter has no influence on the QoE: a higher video bitrate requires high bandwidth without increasing the QoE of the end-users. Therefore, the CVO controller can order the VO of the camera to acquire the video at a low bitrate to save bandwidth. In a more complex scenario, the control module could make further decisions. For instance, if the proposed MIoT vehicle application would allow the end-user application to interact with the vehicle (e.g., to reduce the speed), the controller will have to find a trade-off between synchronization of the different data flows and data presentation delay. Precisely, if the video sequence and the data vehicle are not synchronized, due to network delay, the controller can hide this delay by buffering and synchronizing the data before the displaying on the web IoT application, in order to increase the end-user QoE. However, in this case, the end-user interaction with the application will not be in real time. For this reason, the controller has to find a trade-off between data desynchronization and real-time interaction.

### 5.2. Smart Surveillance Application

As a second use case, we consider a smart surveillance application, which consists of a set of smart video cameras, motion and light sensors placed in specific points of a house or a building. The objective is to monitor the spaces and to automatically detect and signal intrusions.

**Figure 10:**
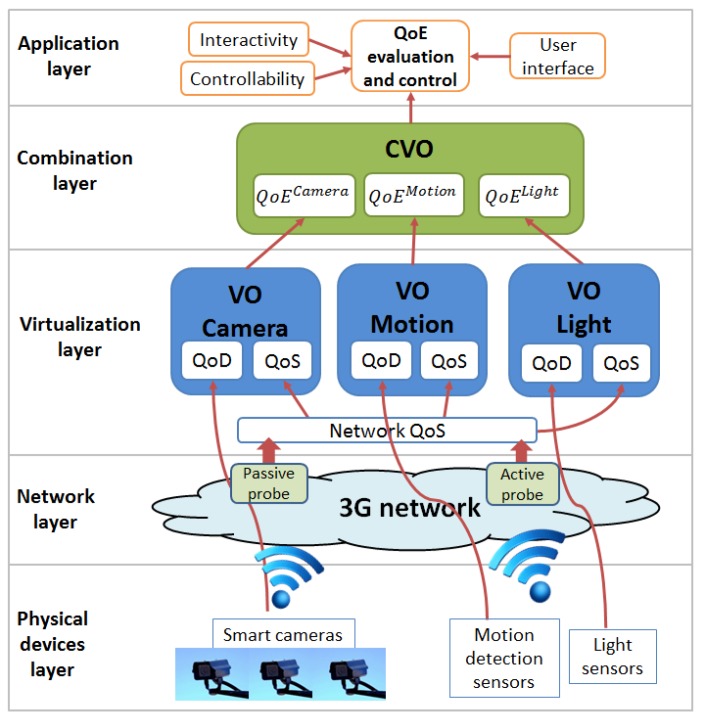
Framework of the smart surveillance application.

Figure 10 shows the proposed QoE framework applied to this specific smart surveillance application. The physical devices are the smart video cameras and the light and motion detection sensors. These devices send the acquired data to their VOs, which have the role of evaluating the quality of the data received. With regard to the camera, objective QoE models for QoE evaluation of video sequences can be used, i.e., models that estimate the QoE as a function of the quality of the recorded videos (bitrate, resolution, codec, i.e., QoD) and of the measured network parameters (packet loss, delay, i.e., QoS). On the other hand, the QoE provided by light and motion detection sensors is highly correlated with their accuracy and fail probability, because the role of these sensors is to detect respectively the presence of light and motion within a space. However, subjective assessments should be conducted to investigate the QoE of the user and to define objective models with regard to these sensors. Specifically, the quality provided by the motion detection sensor may be correlated to its effectiveness in detecting motion of people, as well as ignoring irrelevant motions (e.g., those due to animals living in the house). The quality provided by the light sensor may be correlated to the intensity of the light it is able to detect. For example, it may be important to detect beams of light produced by a flashlight used by a burglar. Moreover, for both sensors, it is important to rapidly send the data to the cloud in order to alert the user as soon as possible. This depends on the QoS of the network and specifically on the latency.

In the cloud, the CVO collects the data provided by the VOs, and the smart surveillance application shows in a web page the video sequences recorded by the video cameras in real time and a history of events created by motion detection and light sensors. Furthermore, alarms can be set to signal whether a motion or a light is detected in specific spaces during the night or when the user is out of the house. When an alarm activates, a message is sent to the user’s smartphone. Moreover, the user is able to access the surveillance application also with his/her smartphone, so that he/she can monitor what is happening if he/she receives a message due to the alarm activation.

The CVO computes the overall QoE provided by the VOs combining the quality of the recorded video sequences with the quality of the light and motion detection sensors. Although the quality of the video frames is important for recognizing people who may enter the house, the quality of the sensors may be even more important because it allows discovering in real time an intrusion. Therefore, the CVO would probably be giving a heavier weight to the quality of sensors in the overall formula for QoE evaluation. However, subjective quality assessments should be conducted to confirm this hypothesis.

Finally, the QoE of the application is evaluated. The factors that determine the QoE in this layer are the user interface and the interactivity and controllability of the application. The user interface is very important because it should be intuitive and easy to use. Accordingly, interactivity and controllability may determine the final opinion of the user. Indeed, although the surveillance system works well (in terms of QoS and QoD), if the user is not comfortable with the utilization of the application (e.g., he/she is not clear how to set the alarms, the way the video sequences are shown is confusing, etc.), this may strongly influence the QoE perceived.

The proposed layered approach allows considering and combining the separate factors that influence the QoE perceived by the user. In this way, it is possible to understand which are the critical influence factors on the QoE, as well as the “weak points” of an application.

## 6. Future Challenges and Conclusions

The Internet of Things encompasses an enormous plethora of applications that will be deployed thanks to the combination of information and services provided by heterogeneous objects, exploiting also opportunistic objects cooperation. These can also be deployed on the fly according to the temporary needs of the users. In this scenario, the evaluation of the QoE becomes even more challenging than what characterizes fixed and well-defined applications scenarios, such as those related to voice and video conferencing. With the proposed layered QoE model, we intend to simplify the evaluation and combination of the contributions of each influence factor to estimate the overall QoE in IoT, with particular attention to multimedia IoT applications. Whereas the proposed methodology gives some directions in this respect, there are key factors that can be obstacles to its use, and there are still challenges to be addressed in any case to make it effective.

One of the major obstacles is the adoption of a reference IoT architecture, which is far from being accepted by the relevant community. Whereas we believe that the composition of simple services will still continue to exist (and then, the relevant combination layer in our model), as it is a key characteristics of also the Internet of Service we have used since at least a decade ago, the role of the key role of the virtual object is still to be proven. In particular, the VO could be divided into different functionalities, some implemented in the physical object and some in the cloud, so that the relevant layer in our model should be split accordingly. As to the challenges, even if trivial, it is worth mentioning that several models are still missing to make the proposal effective. Just to mention one, the QoD for the video signal is still to be defined, and only a few of the existing models can be applied to our case. Additionally, the presence of difference objects also owned by different persons and organization requires that the quality provided by each object and communicated to the VO is reliable, and for this, appropriate actions should be taken to assess the trustworthiness of the objects in this respect.

In this work, we have verified the performance of the proposed QoE model when applied to the use case of an IoT platform aimed at remote monitoring and tutoring practitioners when driving a car. Through subjective evaluations, we have been able to prove that the QoE metrics derived from the layered QoE model are highly correlated with subjective evaluation and allow estimating and controlling the QoE of MIoT applications. However, further investigations are needed, especially with regard to the influence of the video quality on the final QoE. As future work, we also intend to perform experiments with other MIoT applications to verify the model and to introduce further refinements.

## Figures and Tables

**Table 1:** Test Conditions (TC).

TC	Video	Video Bitrate	Accuracy of Vehicle Data	Network Delay	Data Presentation
1	Video 1	1.5 Mbps	100 %	0 s	Analog
2	Video 2	1.5 Mbps	100 %	0 s	Digital
3	Video 3	1.5 Mbps	100 %	3 s	Analog
4	Video 4	1.5 Mbps	100 %	3 s	Digital
5	Video 5	1.5 Mbps	75 %	0 s	Analog
6	Video 6	1.5 Mbps	75 %	0 s	Digital
7	Video 1	700 kbps	100 %	0 s	Analog
8	Video 2	700 kbps	100 %	0 s	Digital
9	Video 3	700 kbps	100 %	3 s	Analog
10	Video 4	700 kbps	100 %	3 s	Digital
11	Video 5	700 kbps	75 %	0 s	Analog
12	Video 6	700 kbps	75 %	0 s	Digital

**Table 2:** Values of the coefficients for the linear regression in Equations (5)–(7).

Coefficient	$a_{1l}$	$K_{1l}$	$a_{2l}$	$K_{2l}$	$a_{3l}$	$K_{3l}$
**Value (digital)**	0.0000	2.5556	0.0483	−1.8750	−0.2153	2.7708
**Value (analog)**	0.0000	2.3056	0.0558	−2.8125	−0.0903	2.3958

**Table 3:** Values of the coefficients for the nonlinear regression in Equations (8)–(10).

Coefficient	$a_{1\mathrm{nl}}$	$K_{1\mathrm{nl}}$	$a_{2\mathrm{nl}}$	$K_{2\mathrm{nl}}$	$a_{3\mathrm{nl}}$	$K_{3\mathrm{nl}}$
**Value (digital)**	1.0000	2.5085	1.0194	0.4140	0.9366	2.5740
**Value (analog)**	1.0000	2.1597	1.0273	0.1829	0.9930	2.1666

**Table 4:** Values of the Pearson correlation between the $MOS_{V}$ values and the quality models defined in Equations (11)–(14) with regard to the digital data presentation.

Quality Model	Equation (11)	Equation (12)	Equation (13)	Equation (14)
**Pearson correlation**	0.97	0.94	0.97	0.95

**Table 5:** Values of the Pearson correlation between the $MOS_{V}$ values and the quality models defined in Equations (11)–(14) with regard to the analog data presentation.

Quality Model	Equation (11)	Equation (12)	Equation (13)	Equation (14)
**Pearson correlation**	0.93	0.87	0.93	0.87
